# *Bupleurum chinense DC.*–Derived Acidic Polysaccharide: Alleviates Lipopolysaccharide-Induced Acute Lung Injury via Activation of Siglec-E

**DOI:** 10.3390/molecules31213989

**Published:** 2026-11-05

**Authors:** Yaoyao Liu, Juanjuan Liu, Jin Li, Hongmei Liu, Xiaodong Ran, Yan Huang, Yi Jia

**Affiliations:** 1 Department of Pharmaceutics, Faculty of Pharmacy and Laboratory Medicine, Army Medical University, Shapingba, Chongqing 400038, China; yaomi987@163.com (Y.L.); lijin0303@126.com (J.L.); hmliu@tmmu.edu.cn (H.L.); rxd1027@163.com (X.R.); 2 Department of Pharmacognosy, Faculty of Pharmacy and Laboratory Medicine, Army Medical University, Shapingba, Chongqing 400038, China; lj1391@163.com

**Keywords:** *Bupleurum chinense DC.*, polysaccharide, Siglec-E, Acute lung injury, neutrophil extracellular traps

## Abstract

Background: Acute lung injury (ALI) is linked to neutrophil extracellular traps (NETs). BP polysaccharides from *Bupleurum chinense DC.* modulate neutrophil function, but their effects on NET formation, release, or degradation are unknown. Purpose: This study tested whether BP-1, an acidic polysaccharide from *Bupleurum chinense DC.*, attenuates NET formation in murine ALI by activating Siglec-E. Methods: BP-1, an acidic polysaccharide isolated from *Bupleurum chinense DC.* and confirmed to bind human Siglec-9 and murine Siglec-E, was physicochemically characterized by SEM, FT-IR, and HPLC. A murine ALI model was induced by intratracheal LPS administration, and the anti-inflammatory effects and molecular mechanisms of BP-1 were systematically evaluated using H&E staining, BALF cytokine analysis, Western blotting, immunofluorescence, and immunohistochemistry. To determine murine Siglec-E dependency, primary neutrophils were isolated from Siglec-E^PKO^ mice and stimulated with nigericin in vitro; NET formation was assessed by immunofluorescence staining. Results: BP-1 contains 81.62% carbohydrates, mainly GalA, Glc, Gal, and Ara. In murine LPS-induced ALI, BP-1 reduced neutrophil infiltration and NET formation—evidenced by lower MPO activity, Cit-H3, and dsDNA in lung tissue and BALF. It also decreased serum IL-1β and TNF-α, and suppressed pulmonary PAD4. In primary neutrophils, BP-1 inhibited nigericin-induced NETs only in the presence of murine Siglec-E; no effect was seen in Siglec-E^PKO^ cells. BLI confirmed that BP-1 binds murine Siglec-E. Conclusion: BP-1 binds Siglec-E and inhibits neutrophil recruitment and NET formation in a mouse model of ALI.

## 1. Introduction

Acute lung injury (ALI) is an acute-onset syndrome with high incidence and mortality [1]. It arises from dysregulated pulmonary inflammation, causing alveolar-capillary barrier disruption—across endothelial and epithelial layers—alveolar epithelial injury, increased vascular permeability, and pulmonary edema, ultimately leading to hypoxemic respiratory failure [2,3]. Standard care targets pulmonary inflammation, typically with glucocorticoids; however, their off-target effects—including hyperglycemia, immunosuppression, and adrenal suppression—limit clinical utility. There is thus an urgent need for lung-selective anti-inflammatory agents that improve efficacy and reduce systemic toxicity. Natural anti-inflammatory compounds from traditional Chinese medicines represent a promising source of candidate agents, and Siglec-9 may serve as a key molecular node linking herbal intervention to the resolution of pulmonary inflammation in ALI.

*Bupleurum chinense DC.*, first recorded in the ‘Shennong Ben Cao Jing’ (Divine Farmer’s Materia Medica), has been used in traditional Chinese medicine (TCM) for antipyretic therapy for ~2000 years. TCM theory attributes its effects to the lung, liver, and gallbladder meridians [4]. It is a core herb in canonical formulae—including Xiao Chai Hu Tang and Si Ni Tang [5]—and served as the principal ingredient in the Lung-Clearing and Detoxifying Decoction (LCDD), widely used during the COVID-19 pandemic in China and Chinese-Canadian communities [6]. Modern studies show that its polysaccharides exert potent anti-inflammatory effects in diverse models, including murine and cellular ALI [7,8,9]. Notably, provides critical preliminary evidence for the present work: their study not only demonstrated anti-inflammatory activity of TCM agents in ALI models but also first established that human Siglec-9 and its murine ortholog Siglec-E can be engaged by polysaccharide constituents derived from TCM [10]. Yet mechanistic understanding remains limited: most evidence comes from crude or partially purified extracts, with few studies identifying direct molecular targets or downstream signaling pathways.

Human Siglec-9 is a canonical inhibitory immune checkpoint receptor in humans and the functional ortholog of murine Siglec-E. Predominantly expressed on neutrophils and monocytes/macrophages, it binds sialylated glycoconjugates and recruits SHP-1/SHP-2 phosphatases via intracellular ITIMs to suppress pro-inflammatory cytokine production and phagocyte activation [11]. Physiological ligands are primarily sialoglycoproteins bearing terminal α2–3 or α2–6 linked sialic acid [12]. Endogenous ligands such as DMBT1 (expressed on endothelial cells), Glycophorin A (on erythrocytes), and MUC5B (secreted by airway epithelium) must multimerize to engage human Siglec-9 with high avidity [13,14]; however, their structural complexity and tissue-restricted localization hinder their development as therapeutic agonists. Fragmented or oxidized hyaluronic acid binds and activates human Siglec-9 without sialic acid [15]. Collectively, these findings establish Siglec-9 as a tractable anti-inflammatory target for lung inflammatory disease. Despite the reported anti-ALI effects of Bupleurum chinense polysaccharides and the capacity of TCM polysaccharides to interact with Siglec-9/Siglec-E, it is unknown whether Bupleurum chinense protects against ALI via Siglec-E-dependent immune modulation.

Building on this, we isolated and characterized the acidic polysaccharide from *Bupleurum chinense DC.* The acidic polysaccharide binds human Siglec-9 and murine Siglec-E, and its preventive efficacy was evaluated in a murine model of lipopolysaccharide-induced ALI.

## 2. Results

### 2.1. BP-1 Physicochemical and Structural Properties

**Figure 1:**
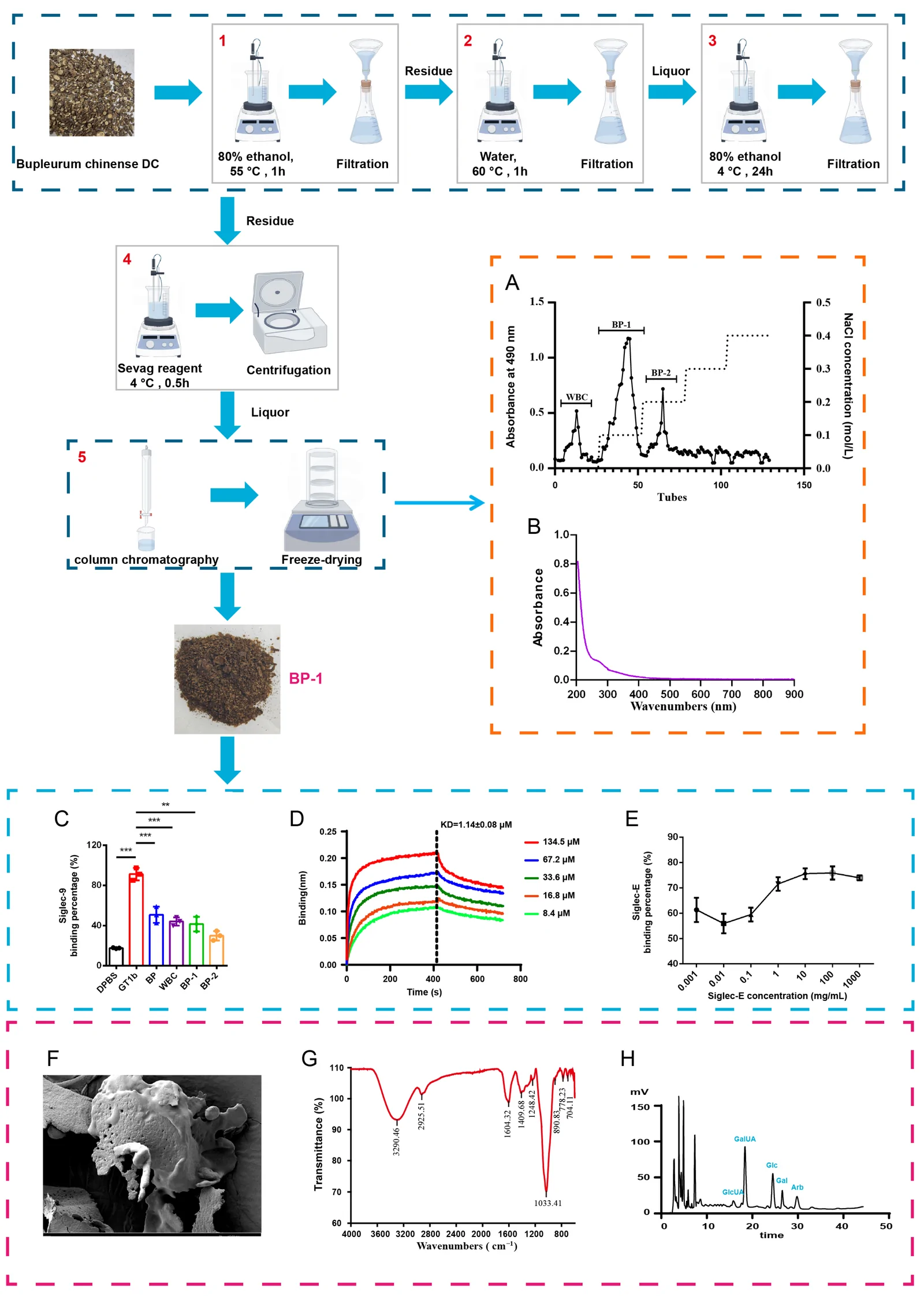
Isolation, purification, functional characterization, and structural analysis of BP-1. (**A**). Elution profile of total polysaccharides purified by DEAE-52 cellulose chromatography; (**B**). UV-Vis absorption spectrum of BP-1; (**C**). Human Siglec-9 binding to bacterial capsular polysaccharides; (**D**). BLI sensorgrams and affinity-fitting curves for BP-1 binding to murine Siglec-E; (**E**). BP-1 and Siglec-E binding affinity; (**F**). SEM images of BP-1 surface morphology; (**G**). FT-IR spectrum of BP-1; (**H**). HPLC chromatogram of BP-1 monosaccharide composition. The results are presented as mean ± SD (n = 3). Statistical analysis was performed using ANOVA, with significance levels indicated as ** *p* < 0.01 and *** *p* < 0.001.

Acidic polysaccharide fractions—WBC (Components of *Bupleurum chinense DC.* were eluted with deionized water), BP-1 (Acidic polysaccharide 1 derived from *Bupleurum chinense DC.*) and BP-2 (Acidic polysaccharide 1 derived from *Bupleurum chinense DC.*)—were isolated from *Bupleurum chinense DC.* via DEAE-52 anion-exchange chromatography (Figure 1A). BP-1, selected for its high isolation yield (51.95% *w*/*w*, Compared with total polysaccharides) (0.0021% *w*/*w*, Compared with *Bupleurum chinense DC.*), was prioritized for all subsequent functional and mechanistic investigations. Compositional analysis revealed a carbohydrate content of 81.62% (*w*/*w*) and an uronic acid content of 40.44% (*w*/*w*), consistent with the structural profile of an acidic polysaccharide; residual protein contamination was below 0.3% (*w*/*w*) (Figure 1B). The content of uronic acid in BP-1 was 40.44%. Scanning electron microscopy (SEM) imaging (Figure 1F) revealed that BP-1 adopts a cloud-like, coiled morphology—indicative of intermolecular aggregation related to non-covalent interactions among surface functional groups. FT-IR spectroscopy (Figure 1G) confirmed its polysaccharide identity: a broad absorption band at 3290 cm^−1^ corresponds to O–H stretching vibrations; a peak at 2925 cm^−1^ arises from C–H stretching; the bands at 1409 cm^−1^ and 1604 cm^−1^ are assigned to asymmetric C=O stretching—characteristic of uronic acid carboxyl groups; peaks at 1033 cm^−1^ are attributable to C–H bending and C–O stretching vibrations, respectively; and the signal at 890 cm^−1^ is diagnostic of β-glycosidic linkages. HPLC monosaccharide analysis (Figure 1H) identified galacturonic acid (GalA), glucose (Glc), galactose (Gal), and arabinose (Ara) in a molar ratio of 32.7: 28.2: 19.1: 5.9. The remaining 14.1% comprises proteins, ash, and uronic acid degradation products from acid hydrolysis. Collectively, these data demonstrate that BP-1 exhibits microstructural heterogeneity.

### 2.2. BP-1 Binds Siglec-9/E

Competitive binding assays demonstrated high-affinity interactions of WBC (The components were eluted with deionized water), total polysaccharides from Bupleurum chinense (BP) and BP-1 (Acidic polysaccharide 1) with recombinant human Siglec-9–Fc (Figure 1C). Biolayer interferometry (BLI) quantification revealed that BP-1 binds murine Siglec-E with higher affinity (KD = 1.14 μg/mL) (Figure 1D). The 1:1 model is only used as an empirical fitting approximation and does not represent the strict monovalent 1:1 molecular stoichiometric binding of BP-1 and Siglec-E. Competitive binding assays further confirmed BP-1 engagement with murine Siglec-E (Figure 1E). Collectively, these findings indicate that BP-1 suppresses neutrophil activation by binding to murine Siglec-E.

### 2.3. BP-1 Attenuated LPS-Induced ALI

**Figure 2:**
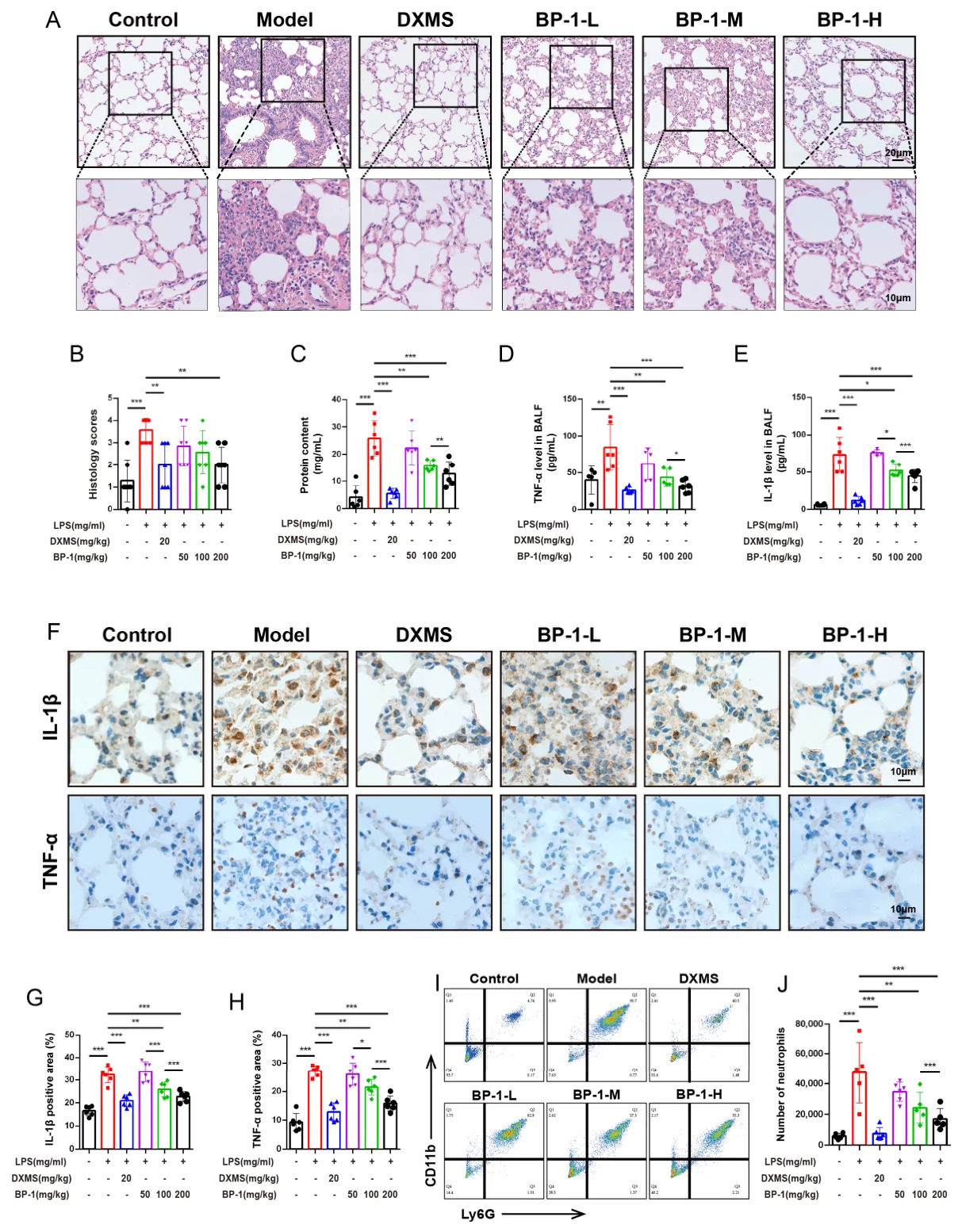
BP-1 demonstrated a protective effect against lung histopathological alterations in mice with LPS-induced (5 mg/kg, i.n.) ALI. (**A**). Representative H&E-stained lung tissue sections; (**B**). ALI severity scores; (**C**). Total protein concentration in BALF; (**D**,**E**). TNF-α and IL-1β levels in BALF, quantified by ELISA; (**F**–**H**). Representative immunohistochemistry images and quantitative analysis of IL-1β and TNF-α in lung tissue; (**I**,**J**). Effects of BP-1 on neutrophil number in BALF. The results are presented as mean ± SD (n = 6). Statistical analysis was performed using ANOVA, with significance levels indicated as * *p* < 0.05, ** *p* < 0.01, and *** *p* < 0.001.

Lung histopathology was evaluated by H&E staining. LPS-challenged mice exhibited severe ALI, characterized by alveolar wall thickening, hemorrhage, and inflammatory cell infiltration—effects significantly attenuated by BP-1 and DXMS (Figure 2A). Relative to vehicle-treated controls, LPS administration markedly increased both histopathological injury scores and BALF total protein concentration; these elevations were dose-dependently reduced by BP-1 treatment (Figure 2B,C). Similarly, LPS induced substantial increases in BALF levels of TNF-α and IL-1β, which were significantly suppressed by BP-1 in a dose-dependent manner (Figure 2D,E). Consistent with these findings, immunohistochemical analysis revealed up-regulated TNF-α and IL-1β protein expression in lung tissue following LPS challenge—changes that were reversed by BP-1, with pronounced efficacy observed at both the high-dose (BP-1-H) and medium-dose (BP-1-M) regimens (Figure 2F–H). Neutrophil accumulation in BALF represents a defining cellular feature of ALI. Flow cytometric quantification confirmed a significant increase in the proportion of Ly6G^+^CD11b^+^ neutrophils in BALF from LPS-induced ALI mice compared with controls, confirming robust pulmonary neutrophil infiltration. BP-1 treatment dose-dependently decreased this neutrophil fraction, with statistically significant reductions observed across all tested doses relative to the LPS-only model group (Figure 2I,J).

### 2.4. BP-1 Reduced Neutrophil Recruitment in LPS-Induced ALI

**Figure 3:**
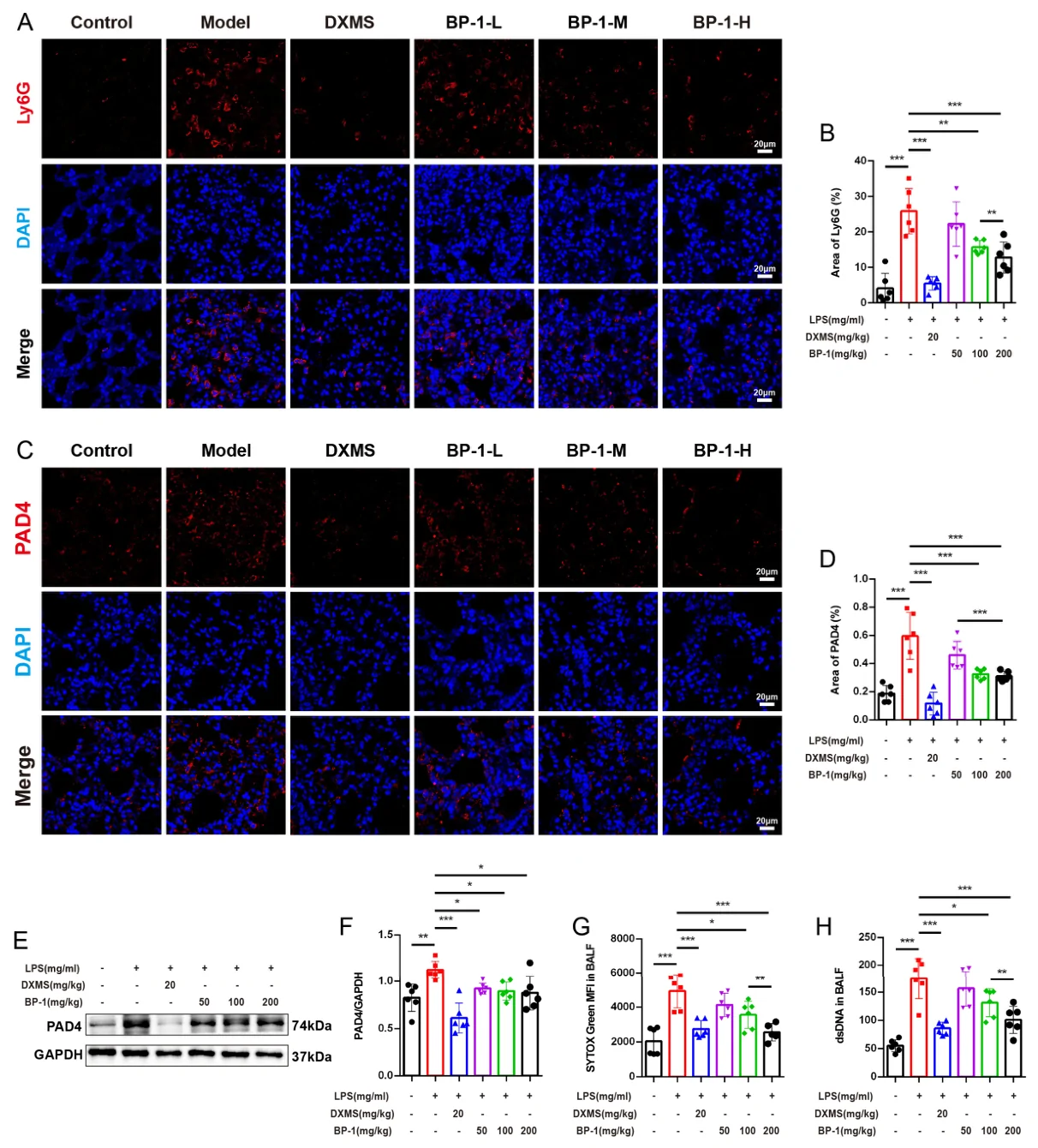
BP-1 significantly attenuated excessive neutrophil recruitment in mice with LPS-induced (5 mg/kg, i.n.) ALI. (**A**,**B**). Representative immunofluorescence images of Ly6G in lung tissue, along with their quantitative analysis; (**C**,**D**). Representative immunofluorescence images of PAD4 in lung tissue, along with their quantitative analysis; (**E**,**F**). Representative Western blot images and quantitative analysis of PAD4 expression in lung tissue, each lane represents an individual biological sample; (**G**,**H**). Extracellular NET formation was assessed by quantifying SYTOX Green-stained extracellular DNA and dsDNA in BALF. The data are expressed as mean ± SD (n = 6), and statistical significance was assessed using one-way ANOVA, with * *p* < 0.05, ** *p* < 0.01 and *** *p* < 0.001 indicating significant differences.

Ly6G immunofluorescence showed pronounced neutrophil infiltration in LPS-challenged lungs—significantly reduced by BP-1 (Figure 3A). Semi-quantification of Ly6G^+^ area confirmed this decrease (Figure 3B). PAD4 staining revealed strong upregulation in LPS-injured lungs, which BP-1 significantly suppressed (Figure 3C). PAD4^+^ area analysis corroborated the suppression (Figure 3D). Western blotting and immunofluorescence, the two independent methods used, confirmed downregulation of PAD4 protein expression following BP-1 treatment (Figure 3E,F). Relative to vehicle-treated controls, LPS challenge significantly increased cell-free NETs and dsDNA in BALF; BP-1 treatment significantly reduced NET levels compared with the LPS-only group (Figure 3G,H).

### 2.5. BP-1 Inhibited MPO and CitH3 in LPS-Induced ALI

**Figure 4:**
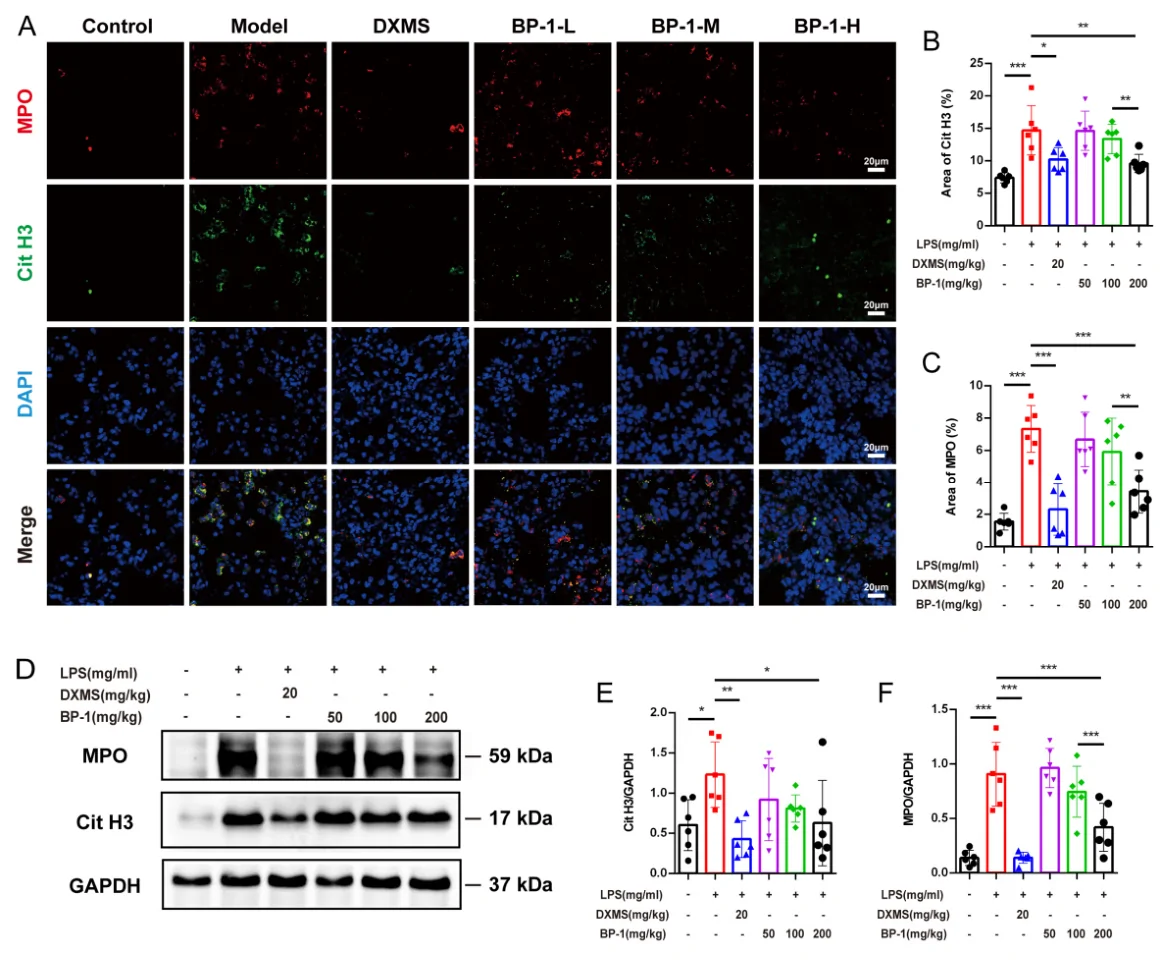
BP-1 suppressed excessive NET formation in mice with LPS-induced (5 mg/kg, i.n.) ALI. (**A**–**C**). Representative immunofluorescence images of MPO and Cit-H3 in lung tissue, together with quantitative analyses. (**D**–**F**). MPO and Cit-H3 expression levels in lung tissue were determined by Western blotting, with quantitative data presented, each lane represents an individual biological sample. The data are expressed as mean ± SD (n = 6), and statistical significance was assessed using one-way ANOVA, with significance levels indicated as * *p* < 0.05, ** *p* < 0.01, and *** *p* < 0.001.

To evaluate the role of NETs in ALI, we quantified MPO and CitH3—established biomarkers of NET formation and release. LPS challenge significantly increased MPO and CitH3 protein levels in lung tissue; BP-1 treatment suppressed all assessed biomarkers, with maximal inhibition achieved at the highest dose tested (Figure 4A–C). Western blotting and immunofluorescence independently confirmed downregulation of both MPO and CitH3 protein expression following BP-1 administration (Figure 4D–F). Collectively, these data demonstrate that BP-1 inhibits MPO and CitH3 protein expression in LPS-induced ALI.

### 2.6. BP-1 Suppresses NET Formation by Engaging Murine Siglec-E

**Figure 5:**
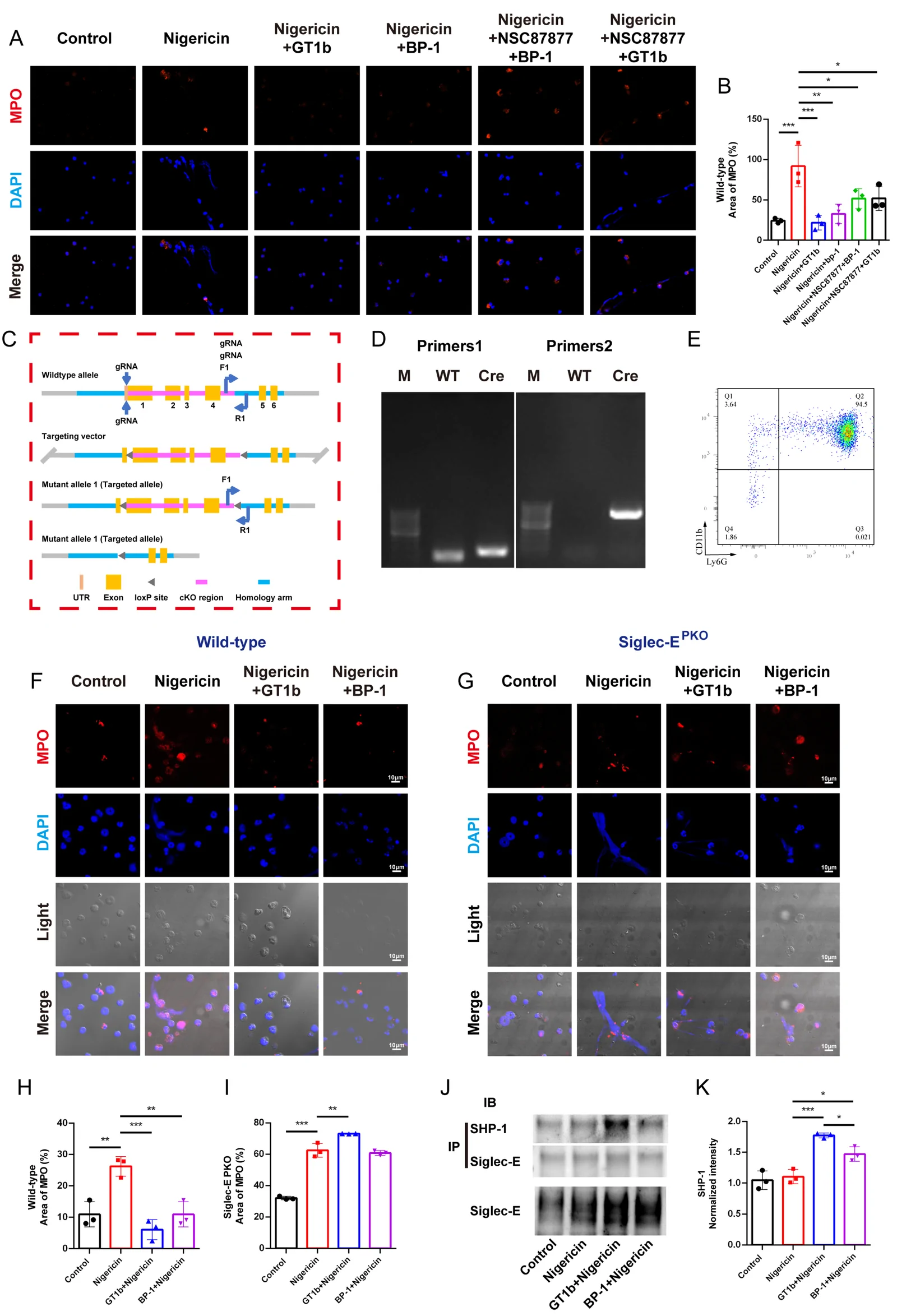
Siglec-E deficiency in neutrophils exacerbates nigericin-induced NET overactivation. (**A**,**B**) BP-1 regulates MPO expression in wild-type neutrophils and associates with SHP-1 (IF + Quantification); (**C**) Siglec-E PKO Mouse Generation Protocol; (**D**) Target Gene Identification in Siglec-E PKO Mice; (**E)** Purity Assessment of Bone Marrow-Derived Neutrophils; (**F**,**H**) BP-1 reduces MPO-associated mean fluorescence intensity in wild-type neutrophils stimulated with nigericin (IF + Quantification); (**G**,**I**) BP-1 did not reduce MPO-associated mean fluorescence intensity in Siglec-E PKO Mice neutrophils stimulated with nigericin (IF + Quantification); (**J**) SHP-1–Siglec-E Interaction: Co-IP from Nigericin-stimulated neutrophils using Anti-SHP-1 beads; (**K**) Western blot Analysis of Co-Immunoprecipitated Complexes, each lane represents an individual biological sample. Data were presented as the mean ± SD (n = 3). Statistics were determined by one-way ANOVA. * *p* < 0.05, ** *p*< 0.01, and *** *p* < 0.001.

Bone marrow-derived neutrophils were isolated from wild-type and Siglec-E^PKO^ mice (Figure 5E). BP-1 regulates MPO expression in neutrophils from wild-type mice and exhibits a functional association with SHP-1 (Figure 5A,B). To determine whether murine Siglec-E is required for this effect, we generated myeloid-specific Siglec-E knockout mice (Siglec-E^PKO^) (Figure 5C,D). Neutrophils from Siglec-E^PKO^ mice exhibited robust nigericin-induced NET formation. Critically, neither BP-1 nor the canonical Siglec-E ligand GT1b suppressed NET formation in nigericin-stimulated neutrophils from Siglec-E^PKO^ mice (Figure 5G,I)—whereas both agents potently inhibited NET formation in nigericin-stimulated neutrophils from wild-type mice (Figure 5F,H). In neutrophils, BP-1 induced tyrosine phosphorylation of the immunoreceptor tyrosine-based inhibitory motif (ITIM) within Siglec-E, as confirmed by co-immunoprecipitation followed by immunoblotting (Figure 5J,K).

## 3. Discussion

Crude polysaccharides from *Bupleurum chinense DC.* attenuate pulmonary inflammation and neutrophil infiltration in murine ALI models [8,16], and the aqueous extract of *Bupleurum chinense DC.* suppresses macrophage activation and pro-inflammatory cytokine secretion [17,18]. However, their chemical heterogeneity obscures precise molecular targets; thus, isolation and identification of bioactive constituents are essential for elucidating anti-inflammatory mechanisms. Beyond *Bupleurum chinense DC.*, polysaccharides from *Arctium lappa*, *Melastoma dodecandrum*, *lignified okra*, and *Bletilla striata* show broad anti-inflammatory activity across diverse disease models [19,20,21,22]. However, most studies report only downstream phenotypic effects without identifying direct protein targets or ligand–receptor interactions. This study demonstrates that BP-1 markedly attenuates LPS-induced severe pulmonary injury in mice, as evidenced by reduced histopathological damage and decreased expression of the pro-inflammatory cytokines TNF-α and IL-1β in both lung tissue homogenates and BALF supernatants. NETs are well-established drivers of ALI pathogenesis [23,24,25]. Yet, no clinically approved therapies specifically target NET formation or clearance. In a murine model of LPS-induced ALI, BP-1 significantly attenuated neutrophil infiltration into the lungs. Concurrently, BP-1 reduced the expression of PAD4 and Cit-H3, as well as MPO activity in lung tissue; moreover, the concentrations of TNF-α, IL-1β, SYTOX Green-stained extracellular DNA, and dsDNA in the BALF supernatant were decreased. In summary, these findings demonstrate that BP-1 alleviates pathological neutrophil infiltration and NET-associated responses in a murine model of LPS-induced ALI.

Notably, human Siglec-9 signaling activation suppresses NET release: synthetic human Siglec-9 agonists significantly reduce SARS-CoV-2–induced NETosis in human neutrophils [26,27], suggesting that pharmacologic targeting of the human Siglec-9 pathway represents a mechanistically grounded strategy to mitigate pulmonary NET accumulation and prevent ALI progression. Human Siglec-9, highly expressed on activated human neutrophils, contains an immunoreceptor tyrosine-based inhibitory motif (ITIM) that recruits and activates the phosphatase SHP-1 upon ligand engagement. This triggers the dephosphorylation of key inflammatory signaling molecules—thereby dampening neutrophil activation [15]. BP-1 binds Siglec-E and promotes its recruitment to SHP-1. BP-1 effectively suppresses nigericin-induced MPO elevation in wild-type mice, this effect was abolished by the SHP-1 inhibitor NSC-87877. In contrast, BP-1 failed to inhibit nigericin-induced MPO elevation in bone marrow–derived neutrophils isolated from Siglec-E KO mice. These findings demonstrate that BP-1 inhibits NETosis in a Siglec-E-dependent manner.

This study has four key limitations. First, BP-1’s structural characterization is incomplete; its configuration remains unresolved. Second, Siglec-E is the murine receptor and Siglec-9 is the human receptor; Siglec-E is the functional homolog of human Siglec-9. BLI assays used only murine Siglec-E [28], limiting translational relevance. Third, phenotypes were assessed in LPS-induced acute sterile inflammation, which does not model chronic or bacterial infection. Fourth, this study lacks pharmacokinetic, tissue distribution and toxicity data for IV-administered BP-1 (50–200 mg/kg). The dose range was based on published studies of similar polysaccharides, but BP-1’s systemic exposure, bio distribution, and safety after IV injection remain unknown. Follow-up PK, tissue distribution, and toxicity studies are needed. Therefore, the efficacy of BP-1 in clinically relevant settings requires further experimental validation.

## 4. Materials and Methods

### 4.1. Major Reagents

*Bupleurum chinense DC.* was purchased from Chongqing Tongjunge Pharmacy Chain Co., Ltd. (Chongqing, China) and authenticated using morphological and microscopic identification in accordance with the Chinese Pharmacopoeia by Prof. Peng Li from Army Medical University; LPS (*Escherichia coli* O127:B8, L3129; Sigma-Aldrich, St. Louis, MO, United States), nigericin sodium salt (HY-100381; MCE, Monmouth Junction, NJ, United States), dexamethasone (IF1010; Solarbio, Beijing, China) and sytox green (AP6325; alphabio, Seattle, WA, United States) were used in cell and animal experiments; TNF-α (ML002095V; Novus Biologicals, Centennial, CO, United States) and IL-1β (ML301814V; Novus Biologicals, Centennial, CO, United States) were used for ELISA detection; The APC-conjugated Ly6G monoclonal antibody (AC0276; Abclonal Technology, Wuhan, China) was used for flow cytometry and immunofluorescence staining; anti-PAD4 antibody (A1906; abclonal, Wuhan, China), anti-MPO antibody (AF3667; R&D, Minneapolis, MN, United States), anti-Cit H3 antibody (ab281584; Abcam, Cambridge, United Kingdom), anti-β-actin antibody (AF0003; Beyotime Biotechnology, Shanghai, China), GAPDH antibody (AF0006; Beyotime Biotechnology, Shanghai, China), HRP-conjugated goat anti-mouse/rabbit IgG (H+L) (9116; Cell Signaling Technology, Danvers, MA, United States), and Alexa Fluor 647 Affinipure goat anti-rabbit IgG (H+L) (111-005-047; Jackson ImmunoResearch, West Grove, PA, United States), were used for Western blotting and immunofluorescence staining; Human Siglec-9 Fc (1139-SL-050; R&D Systems, Minneapolis, MN, United States), murine Siglec-E Fc (5806-SL-050; R&D Systems, Minneapolis, MN, United States), murine Siglec-E antibody (sc-377477; Santa Cruz Biotechnology, Dallas, TX, United States), and SHP-1 antibody (ab227503; Abcam, Cambridge, United Kingdom) were used for binding and immunoprecipitation (IP) experiments.

### 4.2. Extraction and DEAE-Cellulose–Based Purification of BP-1

A total of 500 g of *Bupleurum chinense DC.* decoction pieces were weighed and heated to 55 °C. Pigments were removed by refluxing with 80% ethanol (1:5, *w*/*v*) for 1 h, then filtering while hot. The residue was extracted three times with deionized water (1:5, *w*/*v*, 60 °C, 1 h each). Combined aqueous extracts were concentrated to 500 mL. The concentrate was centrifuged (4000× *g*, 10 min, 4 °C); ethanol was added to the supernatant (80% *v*/*v*) and stored at 4 °C for 24 h to precipitate polysaccharides. The precipitate was collected, air-dried, and lyophilized to give crude polysaccharide. A 100 mg portion of crude polysaccharide was dissolved in deionized water and treated with Sevag reagent (chloroform:isoamyl alcohol = 4:1, *v*/*v*) for 30 min, followed by centrifugation (4000× *g*, 5 min, 4 °C). The protein layer was discarded and the process repeated until no interface remained. The aqueous phase was concentrated and lyophilized to yield purified Bupleurum polysaccharide (BP). BP (20 mg/mL in deionized water) was centrifuged (4000× *g*, 10 min, 4 °C) and loaded onto a DEAE-cellulose column. Elution used a linear gradient of deionized water and 0.5 M NaCl (1 mL/min). Fractions were pooled based on total sugar content (phenol-sulfuric acid assay), dialyzed (3.5 kDa MWCO, 48 h), concentrated, and lyophilized to afford purified fractions.

GalA standards (0, 0.125, 0.25, 0.5, 0.75, 1.0 mg/mL): add 40 μL sulfamic acid and 2.5 mL conc. H_2_SO_4_; mix; boil 20 min; cool to RT; add 40 μL m-hydroxybiphenyl; mix; stand 15 min in dark; measure A_525_. Calibration curve: GalA concentration (x-axis) vs. absorbance (y-axis); linear fit (R^2^ > 0.999). BP-1 (0.1 mg/mL) was processed identically; 200 μL was added to a 96-well plate; A_525_ was measured; uronic acid content calculated as mg GalA eq./mg polysaccharide.

2.0 mg of BP-1 was dissolved in 1.0 mL 2 mol/L HCl–methanol, purged with N_2_, sealed, and incubated at 80 °C for 6 h. The sample was dried under N_2_. Then, 1.0 mL 2 mol/L TFA was added; the sample was purged with N_2_, sealed, and hydrolyzed at 120 °C for 1 h. After cooling to room temperature, methanol was added to precipitate TFA, and the sample was centrifuged at 10,000 *g* for 5 min; supernatant was discarded. This process was repeated 3–5 times. Pellet was redissolved in 1.0 mL ultrapure water. A total of 200 μL was mixed with 400 μL 0.3 mol/L NaOH and 400 μL 0.5 mol/L PMP, and the mixture was derivatized at 70 °C for 1 h. After cooling, the mixture was centrifuged at 10,000 *g* for 5 min; supernatant was collected. pH was adjusted to 7.0–7.1 with 0.2 mol/L HCl. A total of 2.0 mL dichloromethane was added, and the mixture was vortexed and centrifuged at 2000 *g* for 5 min; organic phase was discarded. This process was repeated until dichloromethane layer was colorless. Aqueous phase was dried under N_2_ at 40 °C to near dryness, reconstituted with ultrapure water, filtered through a 0.45 μm nylon membrane, and injected for HPLC analysis. Chromatographic analysis was performed on an Agilent 1200 HPLC system equipped with a Shim-pack GIST C18 column (4.6 mm × 250 mm, 5 μm). The mobile phase consisted of 0.1 mol/L phosphate buffer (pH 6.7) and acetonitrile (82.8:17.2, *v*/*v*) at a flow rate of 1.0 mL/min. Detection was carried out at 254 nm, with the column maintained at 30 °C and an injection volume of 10 μL. Hydrolysates were UV-detected, and monosaccharides were identified and quantified using standard mixtures. For SEM (accelerating voltage of 10 kV), purified BP-1 was dried, homogenized, mounted on conductive tape, and gold-sputtered under vacuum. Surface morphology was imaged at the specified accelerating voltage.

### 4.3. FT-IR Spectroscopy

BP-1 was vacuum-dried overnight prior to FT-IR analysis. Spectra were acquired on a PerkinElmer Spectrum 100 FT-IR spectrometer over the range 4000–400 cm^−1^ with 32 scans at 4 cm^−1^ resolution.

### 4.4. Binding Assay of BPs to Human Siglec-9 and Murine Siglec-E

Human Siglec-9 or murine Siglec-E was pre-incubated with WBC, BP-1, or BP-2 in binding buffer for 1 h at RT. Wells were blocked with 5% BSA for 2 h, washed, then incubated with the complexes for 1.5 h at RT. After three washes, goat anti-human IgG–HRP was added and incubated for 45 min at 37 °C. Following three more washes, HRP substrate was added and absorbance read at 450 nm. BP-1 binding affinity for murine Siglec-E-Fc was measured by BLI using Octet RED96e (Sartorius, PA, USA) and SA streptavidin sensors. Sensors were preconditioned in DPBST (PBS + 0.05% Tween 20) for 10 min, loaded with biotinylated murine Siglec-E FC to ~1 nm shift, then exposed to 2-fold serial dilutions of BP-1 (8.4–134.5 μg/mL) in DPBST. Association and dissociation were recorded and globally fitted to a 1:1 model using Data Analysis Software v9.0.

### 4.5. Animal Experimentation

A total of 48 male C57BL/6J mice (22 ± 2 g) were purchased from Hunan SJA Laboratory Animal Co., Ltd (Changsha, China). and housed under standard conditions (12 h light/dark, 25 °C, 50–60% humidity) with ad libitum chow and water. All procedures followed the NIH Guide for the Care and Use of Laboratory Animals and were approved by the IACUC of Army Medical University. Mice were ear-tagged and randomly assigned to six groups (n = 8 per group): (a) saline control (i.n.), (b) LPS model (5 mg/kg, i.n.), (c) LPS (5 mg/kg, i.n.) + dexamethasone (DXMS, 20 mg/kg, i.p.), (d) LPS (5 mg/kg, i.n.) + BP-1-L (50 mg/kg, i.v.), (e) LPS (5 mg/kg, i.n.) + BP-1-M (100 mg/kg, i.v.), (f) LPS (5 mg/kg, i.n.) + BP-1-H (200 mg/kg, i.v.). BP-1 or saline was given i.v. 30 min before i.n. LPS. At 12 h post-LPS, mice were euthanized by CO_2_ inhalation + cervical dislocation; lungs were excised, weighed, and fixed in 4% paraformaldehyde for histopathology. Two animals per group served as quality controls (QC) and underwent gross pathology and tissue integrity checks to confirm proper sample collection and preservation. They were excluded from statistical analysis because the pre-specified plan required complete primary endpoint data, which they lacked. The remaining six were subjected to formal analysis. Two lungs were harvested per animal: one for histology and immunofluorescence analysis (one non-overlapping microfield randomly selected per section for quantification), the other for Western blotting. All mice were randomly allocated to experimental groups using a random number generator to ensure balanced baseline conditions. All in vivo operations, including LPS modeling and BP-1 administration, were performed by researchers blinded to group assignments. Collected tissue samples were anonymously coded before processing, staining and quantification. Histological staining, image acquisition and quantitative analysis were completed by independent investigators who were unaware of the experimental grouping. Group information was decoded only after all quantitative analyses and histological scoring were finalized to avoid subjective bias.

### 4.6. Histological Analysis

Lung tissues from six animals per group were fixed in 4% paraformaldehyde, paraffin-embedded, and sectioned at 5 μm. H&E and IHC staining were performed, and sections were imaged at 20× magnification. Histopathological changes—alveolar edema, hemorrhage, septal thickening, and leukocyte infiltration—were scored 0–4 by two blinded pathologists [29] scale: 0 = absent; 1 ≤ 25%; 2 = 25–50%; 3 = 50–75%; 4 ࣙ 75% involvement.

### 4.7. Immunofluorescence Analysis of Lung Tissue

Lung tissues harvested 12 h post-LPS were cryosectioned (10 μm) and co-stained for H3Cit (R2/R8/R17) and MPO using Alexa Fluor 594/647-conjugated secondaries. Confocal images were acquired at 40×. Neutrophils were quantified in random Ly6G^+^ fields; NETs were quantified in CitH3^+^/MPO^+^/DNA^+^ fields. Image J 1.52p was used to calculate positive area % per field to assess neutrophil infiltration and markers of NET formation.

### 4.8. BALF Collection and Analysis

BALF was collected from euthanized mice by tracheal instillation and aspiration of 1 mL ice-cold PBS (3×). Samples were centrifuged (400× *g*, 10 min, 4 °C); supernatants were used for BCA protein assay, and pellets were resuspended for flow cytometry of immune cells.

### 4.9. In Vitro Characterization of NET Formation in Neutrophils

Primary neutrophils were isolated from WT and Siglec-E^PKO^ mouse bone marrow using a commercial kit (STEMCELL Technologies, Vancouver, BC, Canada). Siglec-E^PKO^ mice (Lyz2-cre: Siglec-E^flox/flox^, C57BL/6 background) were purchased from Cyagen. Purity (>95%) was confirmed by CD11b/Ly6G flow cytometry. Cells (5 × 10^4^/well) were seeded in black 96-well plates, pre-treated with BP-1 (0.1 mg/mL) or GT1b (10 μM, 30 min) or NCS87877 (0.318 μM), then stimulated with nigericin (250 μM, 5 h) [30]. Changes in NETs were evaluated by quantifying MPO-associated mean fluorescence intensity.

### 4.10. Quantification of Extracellular DNA in BALF and Cell Supernatants with the Quant-iT PicoGreen dsDNA Assay Kit

exDNA in BALF and cell supernatants was quantified using the Quant-iT PicoGreen dsDNA Assay Kit (Thermo Fisher, Waltham, MA, United States). PicoGreen binds dsDNA selectively and is cell-impermeant, ensuring specific detection of extracellular DNA. Fluorescence (Ex/Em = 480/520 nm) was measured on a microplate reader, and concentrations were calculated from a λ-phage dsDNA standard curve (0–1000 ng/mL).

### 4.11. SYTOX Green Staining for exDNA Detection in Mouse BALF Supernatants

BALF supernatants were collected from mice, stained with SYTOX Green dye (1:30,000 dilution), and incubated in the dark on an orbital shaker for 15 min. Subsequently, 100 μL of each sample was transferred to a microplate, and fluorescence intensity was measured using a plate reader (excitation: 488 nm; emission: 523 nm).

### 4.12. Western Blotting

Lung tissues and primary neutrophils were lysed in RIPA buffer with protease/phosphatase inhibitors. Protein concentration was measured by BCA assay. Equal protein amounts were separated on 10% SDS-PAGE gels, transferred to PVDF membranes, blocked with 5% non-fat milk in TBST (2 h, RT), and incubated overnight at 4 °C with primary antibodies (1:1000). After three TBST washes, membranes were probed with HRP-conjugated secondary antibodies (1:10,000, 2 h, RT), developed with ECL reagent, and imaged using a Bio-Rad ChemiDoc XRS+ system. For co-immunoprecipitation, cell lysates were immunoprecipitated with anti-Siglec-E antibody. Immunoprecipitated proteins were resolved by SDS-PAGE and probed by immunoblotting with antibodies against SHP-1 and total Siglec-E. Band intensities were quantified using Image J 1.52p.

### 4.13. Statistical Analysis

Statistical analysis was performed using GraphPad Prism 8 test for multi-group comparisons, followed by Tukey post hoc correction. The n represents biological replicates. Data are presented as mean ± SD. Significance: * *p* < 0.05, ** *p* < 0.01, *** *p* < 0.001 (two-sided).

## 5. Conclusions

These findings indicate that Siglec-E serves as a functionally essential mediator of LPS-induced NET inhibition. These results provide pharmacological validation of Siglec checkpoint activation by *Bupleurum chinense DC.* for targeted remission of acute lung injury and other neutrophil-mediated inflammatory diseases.

## Data Availability

All data generated or analyzed during this study are included in this published article and its Supplementary Information files.

## Supplementary Materials

The following supporting information can be downloaded at: https://www.mdpi.com/article/10.3390/molecules31213989/s1, Figure S1: Binding Affinity of Various Polysaccharides toward Siglec-9; Figure S2: Weight changes of mice in each experimental group.

## Abbreviations

ALI	Acute Lung Injury
NETs	Neutrophil extracellular traps
WBC	Components of *Bulrurum Chinense DC.* were eluted with deionized water
BP	Polysaccharide derived from *Bulrurum Chinense DC.*
BP-1	Acidic polysaccharide 1 derived from *Bupleurum chinense DC.*
BP-2	Acidic polysaccharide 2 derived from *Bupleurum chinense DC.*
HPLC	High-performance liquid chromatography
MPO	Myeloperoxidase
Cit H3	Citrullinated histone H3
dsDNA	Double-stranded DNA
TFA	Trifluoroacetic acid
PMP	1-phenyl-3-methyl-5-pyrazolone
BALF	Bronchoalveolar lavage fluid
BLI	Bio-Layer Interferometry
H&E	Hematoxylin and Eosin
CRD	Carbohydrate recognition domain
WB	Western blotting
PAD4	Protein Arginine Deiminases 4
SEM	Scanning electron microscopy
