# Supplementary material for: Bupleurum chinense DC.–Derived Acidic Polysaccharide: Alleviates Lipopolysaccharide-Induced Acute Lung Injury via Activation of Siglec-E
Source: Molecules. 2026 Nov 5;31(21):3989. doi: 10.3390/molecules31213989 (PMC13765300; doi:10.3390/molecules31213989)
Supplement: Supplementary file 1 [file molecules-31-03989-s001.zip › molecules-4586099-supplementary.pdf]

Supplementary materials

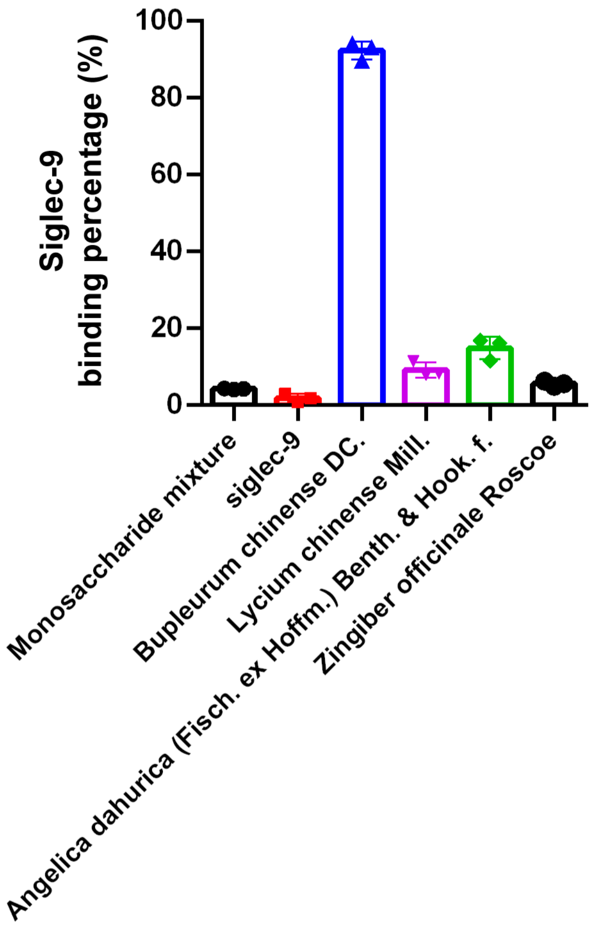

Figure S1. Binding Affinity of Various Polysaccharides toward Siglec-9.

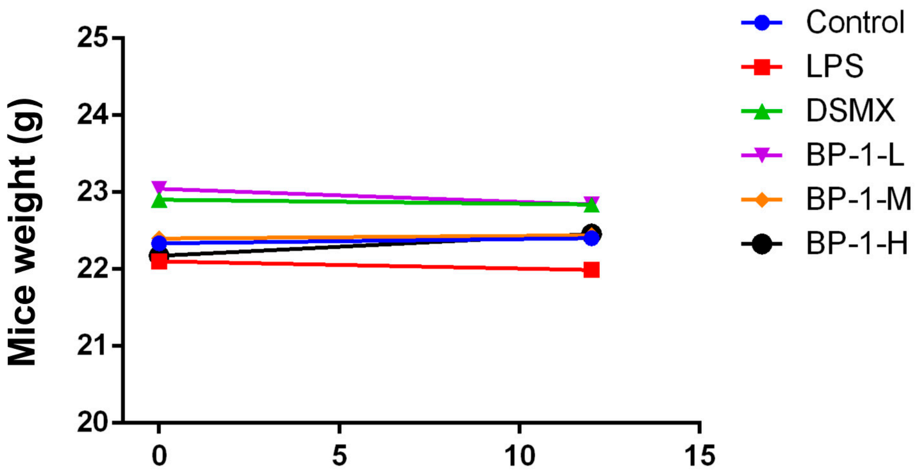

Figure S2. Weight changes of mice in each experimental group.
